# CT-Scan Based Evaluation of Dorsal-to-Ventral Ratios of Paraspinal Musculature in Chondrodystrophic and Non-chondrodystrophic Dogs

**DOI:** 10.3389/fvets.2020.577394

**Published:** 2020-11-04

**Authors:** Katinka Hartmann, Pia Düver, Stephan Kaiser, Carolin Fischer, Franck Forterre

**Affiliations:** ^1^Division of Small Animal Surgery, Department of Clinical Veterinary Medicine, Vetsuisse Faculty, University of Bern, Bern, Switzerland; ^2^Fachtierarztpraxis Am Erzberg, Braunschweig, Germany; ^3^Veterinary Specialists Ireland, Clonmahon, Summerhill, Ireland

**Keywords:** canine, cervical spine, paraspinal musculature, morphometry, biomechanics, intervertebral disk, computed tomographic imaging

## Abstract

**Objective:** To assess and objectively quantify, with CT-scan exams, differences in cervical paraspinal musculature and vertebrae angulation that might influence the different predisposed sites for intervertebral disk disease observed in chondrodystrophic and non-chondrodystrophic breeds.

**Sample:** Retrospective evaluation and analysis of cervical spine CT-scans performed on 30 dogs presented for clinical reasons unrelated to a cervical disk problem. 15 chondrodystrophic (Dachshunds) and 15 non-chondrodystrophic dogs (Labrador Retrievers) were included.

**Procedures:** Height measurements of dorsal and ventral paraspinal musculature were performed on sagittal CT-scan reconstructions to generate dorsal-to-ventral height ratios. Additionally, disk angulation to the floor of the vertebral canal was determined for each cervical disk. On transverse plane images the areas of the dorsal and the ventral paraspinal musculature were measured and ratios calculated. Furthermore, estimations of moments exerted on the disk were evaluated through calculation of a dorsal-to-ventral ratio of moments applied at the level of each disk.

**Results:** Dachshunds showed a relatively more prominent dorsal paraspinal musculature than Labrador Retrievers with statistically significant higher dorsal-to-ventral height ratios at C3/C4, C4/C5, C7/T1 (*p* = 0.034^*^, *p* = 0,004^**^, *p* = 0.004^**^) and a dorsal-to-ventral area ratio at C3/C4 (*p* < 0.001^**^). Regarding the disk angle to the spinal canal floor along the cervical spine, Labrador Retrievers had a less steep conformation compared to Dachshunds with a significant difference at C2/C3 (*p* < 0.001^**^). Relation of moments calculations revealed statistically significant differences at C2/C3 (*p* = 0.021^*^).

**Conclusion and Clinical Relevance:** Significant differences have been found in the cervical spine of chondrodystrophic and non-chondrodystrophic dogs, regarding paraspinal musculature height and area ratios along with ratio of moments and vertebrae angulation. These differences may affect the anatomical and biomechanical dorsal-to-ventral paraspinal muscle relationship and potentially influence the load on intervertebral disks, especially in the upper cervical spine. Our findings could play a role in understanding the development of intervertebral disk disease.

## Introduction

Intervertebral disk disease (IVDD) is a widespread condition in dogs. Chondrodystrophic (CD) and non-chondrodystrophic (NCD) dogs display significant differences in the type, age of onset, prevalence, and spinal location of IVDD. CD dog breeds are characterized by an accelerated form of intervertebral disk (IVD) degeneration at an early age (1). Consequently, the nucleus pulposus (NP) abruptly loses its hydraulic function, with consequent degenerative changes in the annulus fibrosus (AF) (1). In NCD dogs, degeneration of the IVD occurs more gradually later in life (2, 3). Degeneration of the AF can occur independently of NP degeneration, and mainly consists of partial ruptures of the AF fibers (1). Due to these distinct differences, it is conceivable that some etiological factors for IVDD are different between these two groups of dogs.

Causative factors for the high susceptibility of IVDD at specific spinal levels (cervical and thoracolumbar spine) in dogs are still unclear. In CD breeds especially, the cranial aspect (C2/C3) of the cervical spine is found to be at highest risk for cervical IVDD (4, 5). Non-chondrodystrophic (NCD) breeds, such as Labrador Retrievers, are more commonly affected in the caudal cervical spine, mainly the sixth (C6) to seventh (C7) intervertebral disk space (1, 5–8). It has been proposed that the transition from a rigid to a more flexible spine segment is a causative biomechanical factor for IVD pathologies in CD dogs; however, definitive evidence to support this theory, especially for the cervical spine, is still to be found (9).

The cervical spine can be regarded mechanically as a series of freely-hinged vertebrae needing further support from surrounding tensile structures to control posture. The intervertebral disk together with the adjacent cervical vertebrae, respective facet joints, ligaments, and musculature form a motion segment. Within these motion segments, the IVDs are bonded cranially and caudally to the adjacent vertebral bodies (10). Therefore, vertebral bodies and their conformation influence the angulation of the disk.

During the different forms of locomotion: axial torsion, flexion-extension, and lateral bending, moments are important loads that modulate the spine. Tensile structures such as muscles and ligaments must be active to compensate for the applied moments, leaving the spine under considerable axial compression (11). The other mechanical loads are transformed by the surrounding musculature into axial compression, and eventually facet joint loads (12). The IVD can be regarded as a water-filled cushion that mediates and transmits compressive forces between vertebral bodies, and that provides mobility, as well as stability, to the spinal segment (11, 13–15).

The paraspinal musculature is part of the complex axial musculoskeletal system with multiple functions (16). The dorsal and ventral musculature components play an essential role in locomotion, dynamic stabilization and they ensure the integrity of the spine (16). In terms of statics, the cervical spine functions as a kinematic chain (17) with the neck and head cantilevering beyond the forelimb, and acting as a reverse bow in relation to the trunk. The musculature of the neck, in combination with the nuchal ligament, provides the tension of the dorsal side in the bridge-arch model described by Fischer (18). An isolated contraction of the dorsal paraspinal musculature, due to increased distance to the geometric center of the disk would rather physically lead to compression of the dorsal and tension on the ventral AF, whereas in contrast an isolated contraction of the ventral paraspinal musculature would rather act the other way around. From a biomechanical point of view a stronger developed muscle group might exert a stronger moment on the disk compared to the antagonist muscle group. The real situation might be more complex in which both muscle groups interact with each other. The maximal force assembled by a muscle depends on muscle morphology such as the physiological cross-sectional area (PCSA), muscle fascicle length, muscle insertion site (19), and muscle fiber quality. In an attempt to evaluate the structural and functional anatomy of the canine neck musculature, a canine cadaveric study of mixed-breed dogs was conducted (20). The authors provided a systematic description of the anatomy and morphometry of the canine neck musculature. Beyond the fact that this information was achieved post-mortem, no comparison was made between CD and NCD dogs. *In vivo* studies to determine muscle forces, loads and stresses in vertebrae are technically difficult to perform and the data obtained from such studies are limited (20). To the author's knowledge, no biometric study has currently addressed the influence of cervical musculature on the different predisposition sites for cervical disk disease in CD and NCD dogs.

The purpose of this study was to evaluate and to compare the morphometry of cervical dorsal to ventral paraspinal muscles and cervical vertebrae angulation in CD and NCD dogs. We hypothesized that there are breed-associated regional differences comparing the dorsal and ventral paraspinal cervical musculature and different cervical vertebrae angulations between chondrodystrophic and non-chondrodystrophic dogs.

## Materials and Methods

### Patients

CT examinations performed between 2012 to 2018, on 15 Dachshunds (CD breed) and 15 Labrador Retrievers (NCD breed) were retrospectively evaluated in the study. All scans were performed, for reasons unrelated to this study, at a small animal referring center on dogs clinically free of cervical spine pathologies. Exclusion criteria were cervical spinal cord compression, pathologies like cervical spondylomyelopathy, congenital cervical vertebral anomalies, incorrect positioning, and poor image resolution.

### CT-Scan Exam

All dogs were anesthetized. A short stay intravenous catheter was inserted into a cephalic vein. Intravenous anesthesia was induced with propofol (4 mg/kg, Narcofol, 10 mg/ml, CP Pharma GmbH, Germany) and diazepam (0.5 mg/kg, Ziapam, 5 mg/ml, Ecuphar, Belgium) titrated to effect. After endotracheal intubation, anesthesia was maintained with isoflurane (1–3 Vol%, Isofluran, CP Pharma GmbH, Germany), oxygen and room air. Dogs were positioned in a standardized dorsal recumbency with their front limbs fully extended cranially (Picture 1). For image acquisition a 16-slice high-powered GE CT BrightSpeed scanner was used (GE Healthcare GmbH, Germany). Slice thickness was set as 0.6 mm at a regular reconstruction interval of 0.4 mm. A soft-tissue window (W 400; L 30) for better visualization and identification of muscular margins was used.

**Picture 1:**
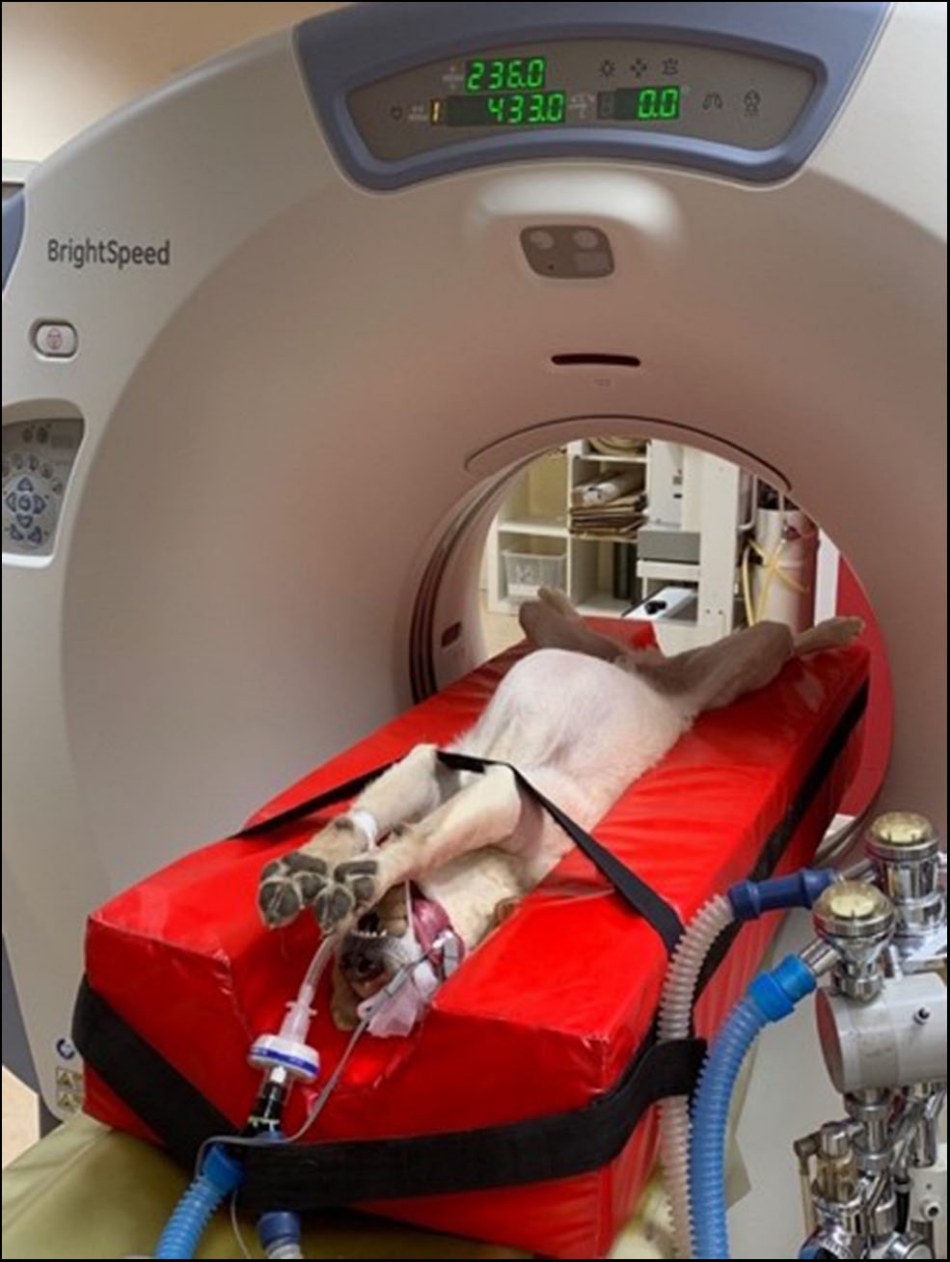
Picture showing anesthetized dog in standard dorsal recumbency with extended front limbs.

### Image Review and Measurements

All measurements were performed by one observer from C2/C3–C7/T1 on sagittal reconstructions and transversal images. Evaluation was performed in the vertical axis of each cervical IVD space. For image review and measurements ADW 4.0 Workstation, GE Healthcare GmbH, Germany was used.

#### Sagittal Plane

##### Height ratio

**Figure 1:**
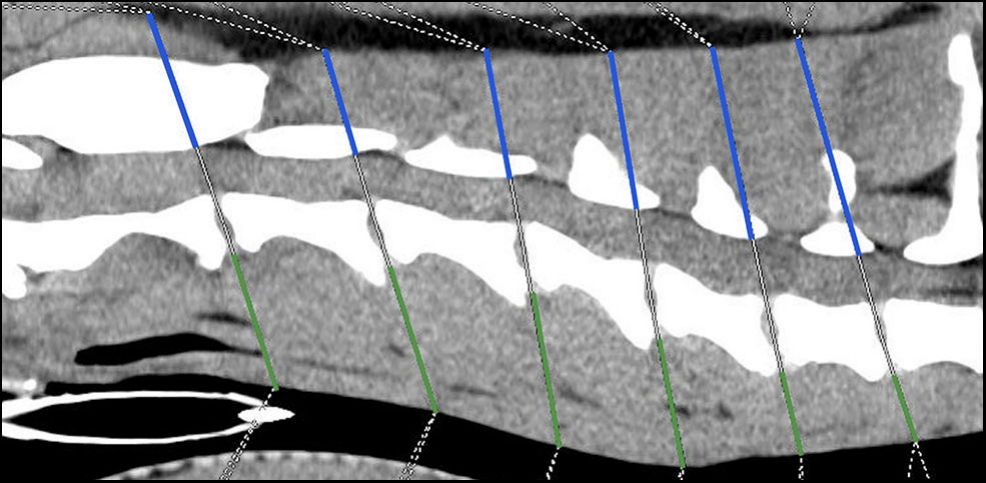
Sagittal plane computed tomographic image; Height measurements, Distance v = green line, Distance d = blue line.

The following two anatomical distances were evaluated at the level of each intervertebral disk space (Figure 1):

Distance *v* Evaluation of the ventral paraspinal musculature: Dorsal tracheal margin to ventral margin of IVD
Distance *d* Evaluation of dorsal paraspinal musculature: To include the paraspinal musculature located dorsal and lateral to the Proc. spinosus and arcus vertebrae, the distance from the ventral margin of arcus vertebrae to the dorsal paraspinal musculature-fat transition was defined. Due to anatomical circumstances at the level of C2/C3 the dorsal border was the dorsal margin of the M. rectus capitis dorsalis major.

The height ratio between the dorsal and ventral paraspinal musculature was calculated using the following equation:

$$\begin{array}{l} Height\;ratio\;of\;paraspinal\;musculature\\ \;=\frac{Distance\;d\;\left(dorsal\;paraspinal\;musculature\right)}{Distance\;v\;\left(ventral\;paraspinal\;musculature\right)} \end{array}$$

For each measurement, either in non-chondrodystrophic or chondrodystrophic breeds, the mean distances were calculated and consequently the mean ratio for each investigated cervical site was determined.

Ratios were determined as a convenient mechanism to express the magnitude of a relative change.

### Angle Measurements

**Figure 2:**
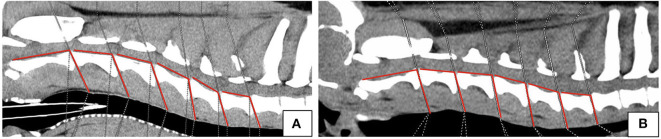
Sagittal plane computed tomographic images of Labrador Retriever **(A)** and Dachshund **(B)** showing angle measurements.

In addition, the angles between the IVD spaces, in relation to the axis of the vertebral column, were measured along the cervical spine in order to evaluate disk angulation (Figure 2). Therefore, a line was drawn on the floor of the vertebral canal from the cranial to the caudal endplate of each vertebral body. A second line was drawn from the caudal endplate in the same axis of the intervertebral disk space to the dorsal margin of the trachea. The angle between those two lines was determined at each cervical intervertebral disk space. For both breeds the mean angle for each location per breed was calculated.

#### Transverse Plane

##### Area ratio

**Figure 3:**
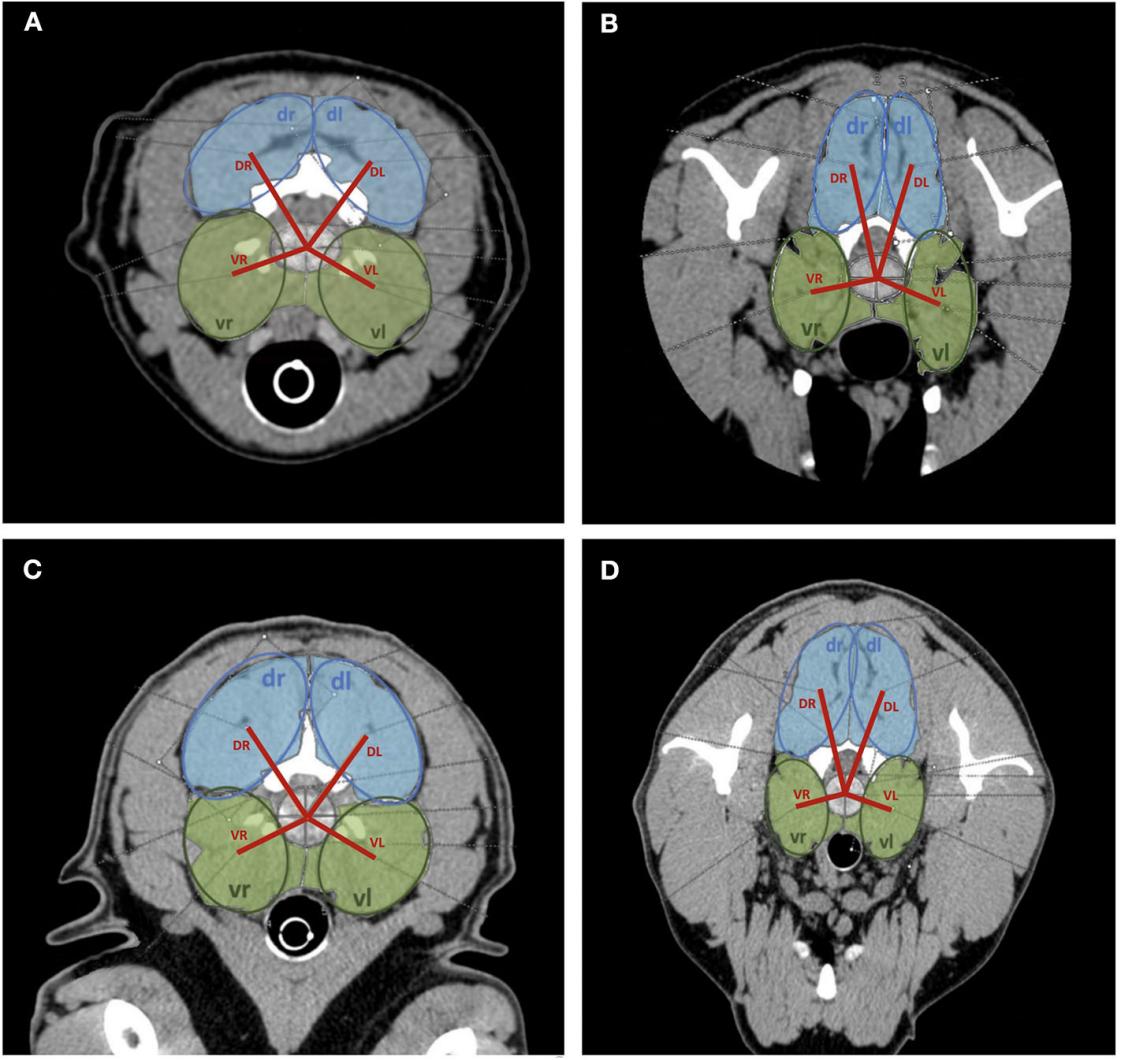
Transversal plane CT images of cervical spine with area measurements of dorsal (light blue) and ventral (light green) paraspinal musculature. Model measurements indicated (dark blue circles = dorsal area measurements; dark green circles = ventral area measurements; red line = distance measurements from the hypothetical center of the intervertebral disk to the center of respective muscle group). **(A)** C2/C3 Dachshund, **(B)** C6/C7 Dachshund, **(C)** C2/C3 Labrador Retriever, **(D)** C6/C7 Labrador Retriever.

In the transverse plane, measurements were taken in the axis of IVD space, at the same level of measurements performed in the sagittal plane. Muscle anatomy varies along the cervical spine, and the muscle groups evaluated were defined for each investigated segment (Table 1). The area of the dorsal and ventral paraspinal musculature was outlined on the CT image and the computer program calculated the value. The sum of the dorsal and ventral paraspinal musculature area from right and left was calculated using ventral right (vr) + ventral left (vl) and dorsal right (dr) + dorsal left (dl) area measurements (Figure 3). The area ratio between the dorsal paraspinal musculature (drl) and ventral paraspinal musculature (vrl) was calculated using the following equation:

$$\begin{array}{l} Area\;ratio\;of\;paraspinal\;musculature\\ \;=\frac{Dorsal\;paraspinal\;musculature\;area\;\left(drl\right)}{Ventral\;paraspinal\;musculature\;area\;\left(vrl\right)}. \end{array}$$

**Table 1:** Considered dorsal and ventral paraspinal muscle groups for each cervical IVD space (10, 21–24).

C2/C3	Dorsal	M. obliquus capitis caudalis M. complexus M. longissimus atlantis et. M. longissimus capitis
	Ventral	M. longus colli M. longus capitis Ventral parts of Mm. intertransversarii
C3/C4	Dorsal	M. obliquus capitis caudalis M. longissimus atlantis et. M. longissimus capitis M. biventer cervicis
	Ventral	M. longus colli M. longus capitis Ventral parts of Mm. intertransversarii M. omotransversarius
C4/C5	Dorsal	M. spinalis cervicis M. biventer cervicis M. multifidi M. complexus M. longissimus capitis et. M. longissimus cervicis
	Ventral	M. longus colli M. longus capitis Ventral parts of Mm. intertransversarii
C5/C6	Dorsal	M. multifidi M. spinalis cervicis M. complexus M. biventer cervicis M. longissimus capitis et. M. longissimus cervicis
	Ventral	M. longus colli M. longus capitis Ventral parts of Mm. intertransversarii M. scalenus medius
C6/C7	Dorsal	M. multifidi M. spinalis cervicis M. complexus M. biventer cervicis M. longissimus capitis et. M. longissimus cervicis Dorsal parts of Mm. intertransversarii
	Ventral	M. longus colli M. scalenus medius
C7/T1	Dorsal	M. multifidi M. spinalis cervicis M. complexus M. biventer cervicis M. longissimus capitis et. M. longissimus cervicis Dorsal parts of Mm. intertransversarii
	Ventral	M. longus colli

### Ratio of Moments

The mean ratio of dorsal-to-ventral moments at each cervical intervertebral disk space was calculated for Labrador Retrievers and Dachshunds. To bundle the two pairs of dorsal and ventral paraspinal musculature left and right, four ellipsoid models were applied over the respective areas. This model was used for the identification of the center of the assumptive muscle group. The distances from these assumptive centers to the center of the disk were measured (VR = ventral right, VL = ventral left, DR = dorsal right, DL = dorsal left) describing the lever arms of each muscle group. Lever arms multiplied with the respective acting forces form the moments (Figure 2). For simplification during calculation, it was assumed that the force of a muscle is proportional to its area. The forces create a moment about the respective IVD. The relation of these moments is defined in the following equation:

$$\begin{array}{l} Relation\;of\;moments=\frac{\left(dr\times \;DR\right)+\left(dl\times DL\right)}{\left(vr\times VR\right)+\left(vl\times VL\right)}. \end{array}$$

### Statistical Analysis

To test the differences between the two groups of dogs at each cervical intervertebral disk space, a two-sided *t*-test was applied, since the data is continuous and approximates a normal distribution. The data were tested for normality using histograms. No concerns of violations of the normality assumptions were raised. Although the sample size is relatively small *n* < 30, the *t*-test is robust enough to still be accurate (for a sample size of 15 in each group) to show a trend in the data. Due to the exploratory nature of the analyses, the presented *p*-values are raw unadjusted *p*-values. *P*–values α <0.05 were considered statistically significant and are flagged with a^*^. Although the analysis is exploratory, multiple comparisons are considered using a Bonferroni adjusted α <0.0083 with a flag of ^**^.

## Results

**Figure 4:**
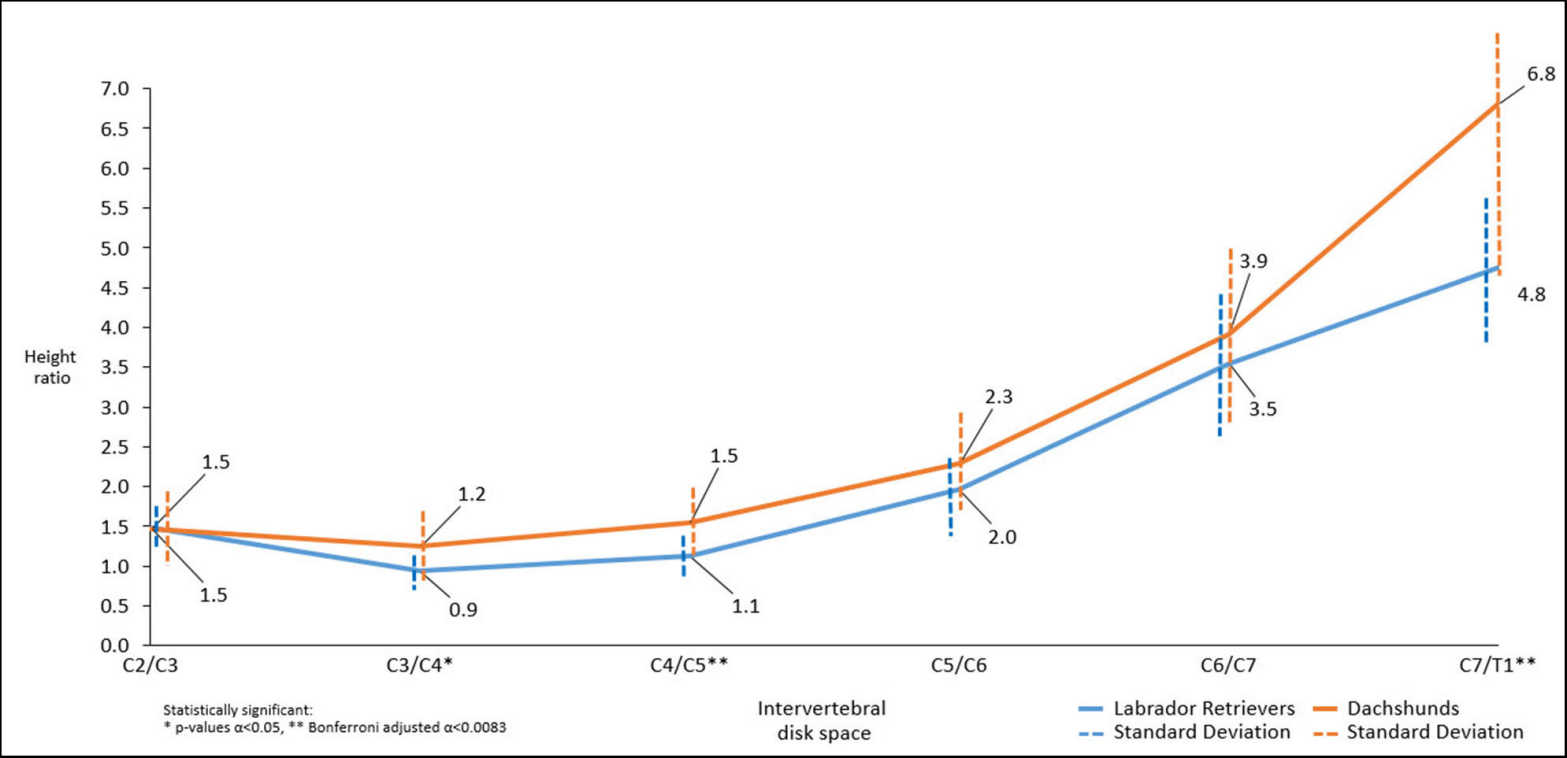
Line graph showing mean dorsal-to-ventral paraspinal musculature height ratio along cervical spine of Dachshunds (orange) and Labrador Retrievers (blue) measured in sagittal plane computed tomographic images. For accurate presentation, measurements pictured are rounded to one decimal place.

**Figure 5:**
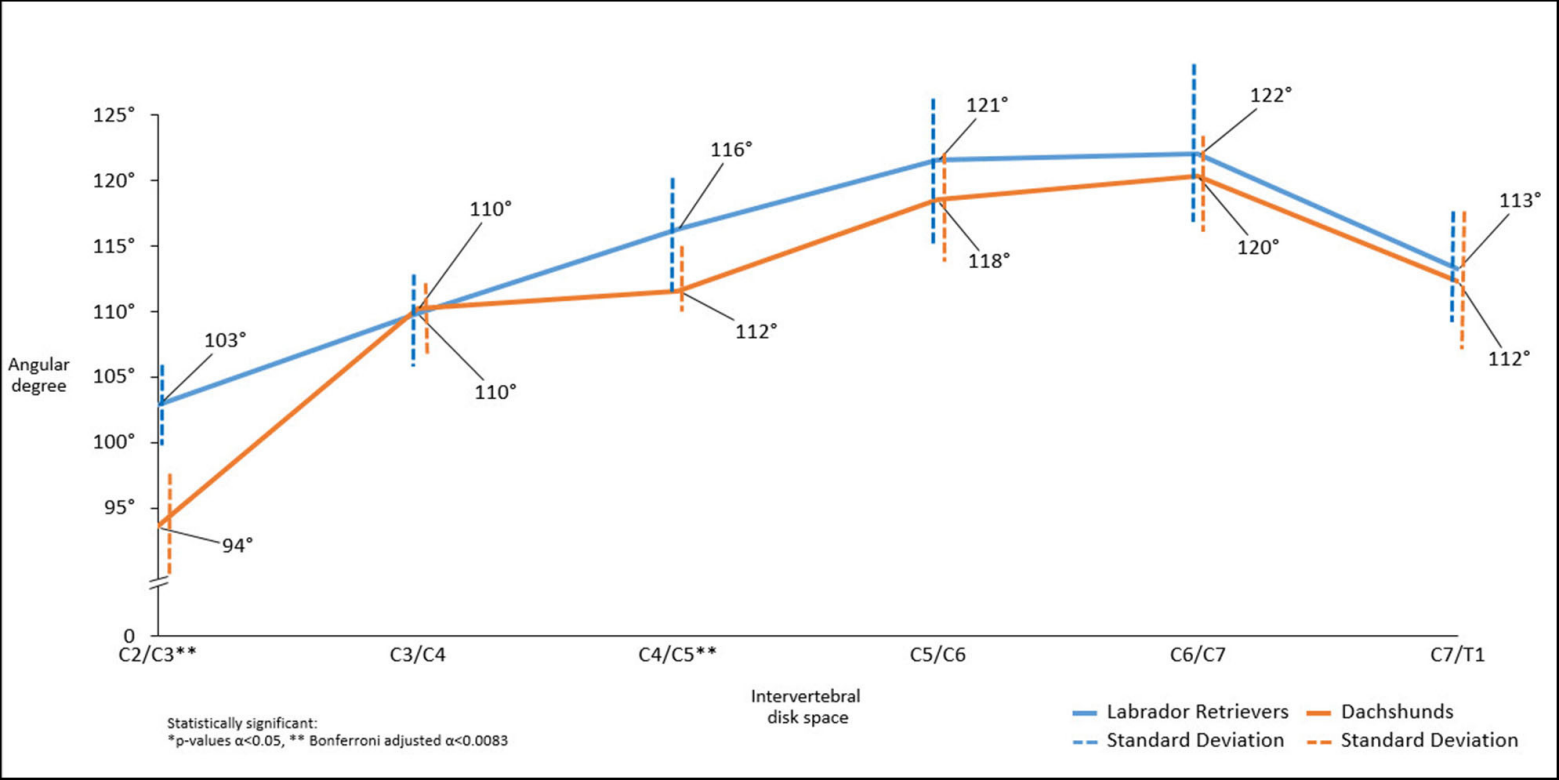
Line graph showing mean angles between IVD space in relation to the axis of the vertebral column along the cervical spine obtained in sagittal plane in computed tomographic images, Dachshunds (orange) and Labrador Retrievers (blue).

**Figure 6:**
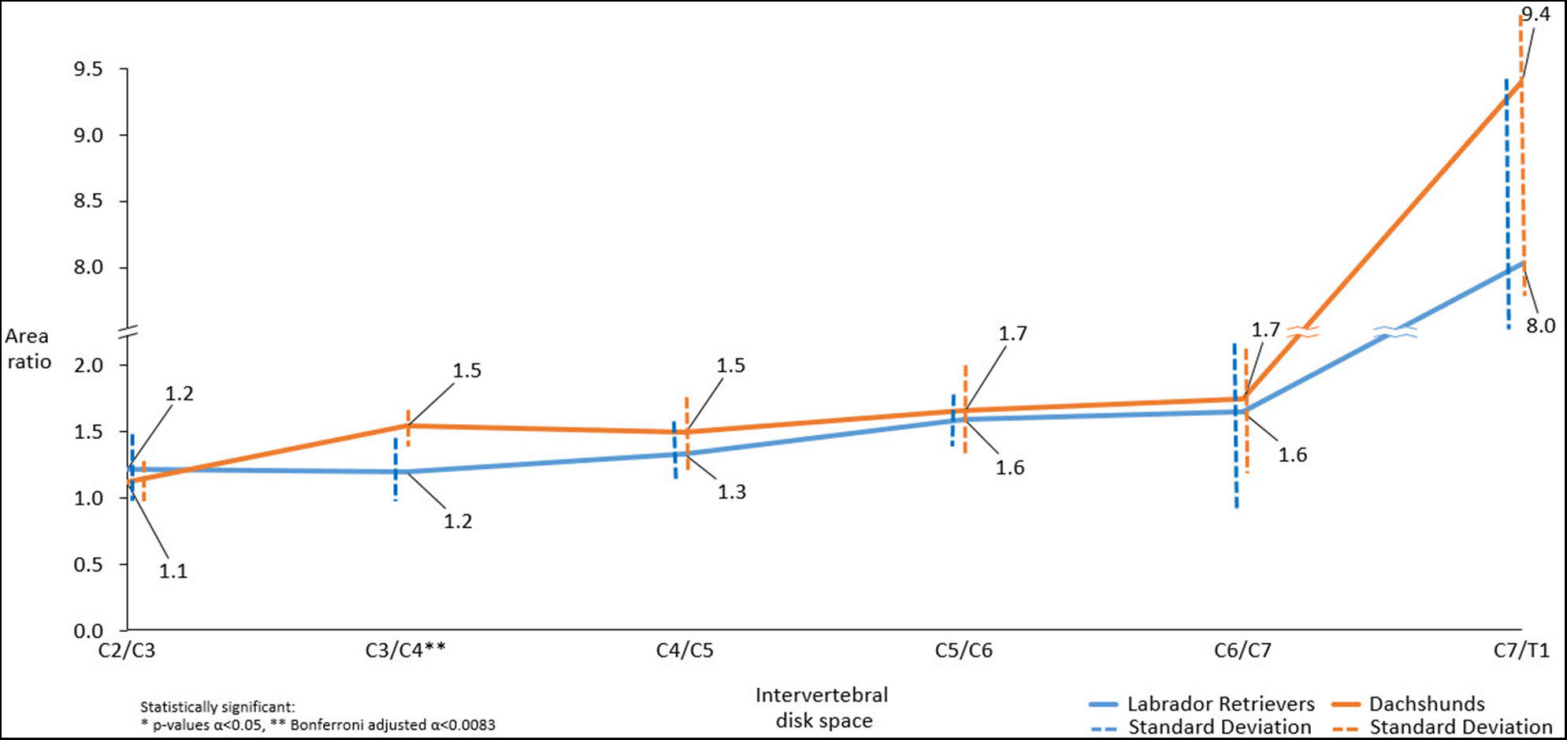
Line graph showing mean dorsal-to-ventral paraspinal musculature area ratio along cervical spine of Dachshunds (orange) and Labrador Retrievers (blue) measured in transverse computed tomographic images. For accurate presentation, measurements pictured are rounded to one decimal place.

**Figure 7:**
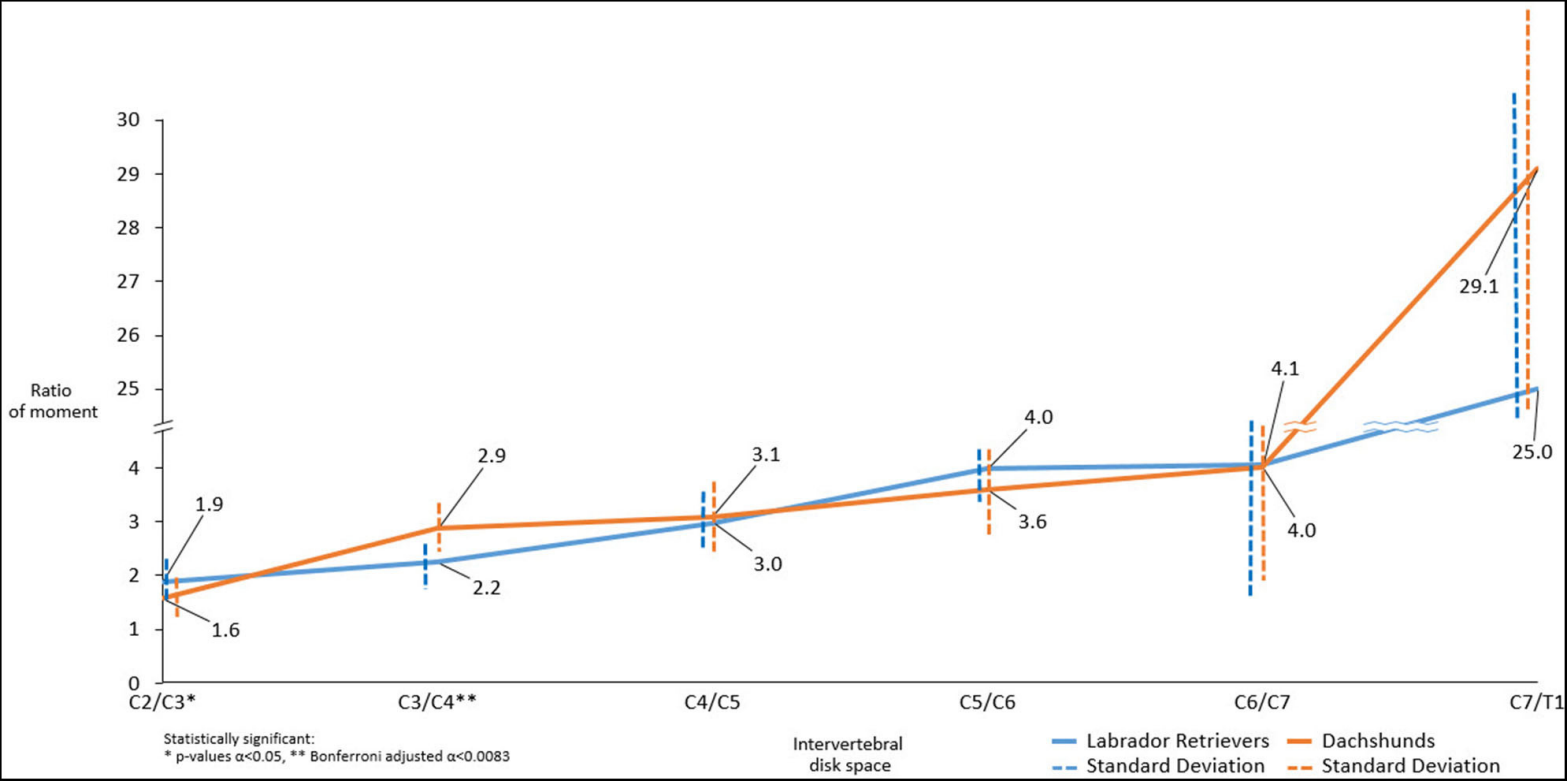
Line graph showing mean ratio of moments determined along cervical spine of Dachshunds (orange) and Labrador Retrievers (blue) measured in transverse computed tomographic images. For accurate presentation, measurements pictured are rounded to one decimal place.

Cervical CT-scans of a total of 30 patients, 15 Dachshunds (CD breed) and 15 Labrador Retrievers (NCD breed) were evaluated. Dachshunds had a mean age of 6.5 years (SD ± 2.47, CI 5.22–7.72) with a mean weight of 8.39 kg (SD ± 2.67). Labrador Retriever had a mean age of 3.7 years (SD ± 2.97, CI 2.23–5.24) and a mean weight of 33.32 kg (SD ± 5,81). An overview of all measurements is presented in Figures 4–**7** and Table 2. The ages between the two groups of dogs were comparable as the confidence intervals (CI) cross each other.

**Table 2:** Mean and standard deviations of Dachshunds (D) and Labrador Retrievers (L) for the four endpoints at each location.

		**Height ratio**			**Angles**			**Area ratio**			**Ratio of moment**		
		**Mean**	**(SD)**	***t*-test**	**Mean**	**(SD)**	***t*-test**	**Mean**	**(SD)**	***t*-test**	**Mean**	**(SD)**	***t*-test**
				***p*-value**			***p*-value**			***p*-value**			***p*-value**
L	C2/C3	1.49	0.30	0.895	103.00	2.89	<0.001**	1.22	0.26	0.222	1.87	0.30	0.021*
D		1.47	0.47		94.00	4.08		1.11	0.17		1.57	0.36	
L	C3/C4	0.94	0.23	0.034*	110.00	3.37	0.730	1.20	0.16	<0.001**	2.24	0.42	0.001**
D		1.25	0.45		110.00	2.78		1.54	0.15		2.86	0.47	
L	C4/C5	1.13	0.28	0.004**	116.00	4.60	0.003**	1.33	0.22	0.106	2.97	0.65	0.643
D		1.54	0.40		112.00	2.39		1.49	0.30		3.09	0.67	
L	C5/C6	1.96	0.46	0.119	121.00	6.09	0.122	1.59	0.19	0.477	3.98	0.42	0.107
D		2.29	0.61		118.00	3.48		1.66	0.30		3.60	0.74	
L	C6/C7	3.55	0.88	0.332	122.00	5.31	0.347	1.64	0.72	0.680	4.06	2.60	0.964
D		3.91	1.06		120.00	3.86		1.75	0.56		4.02	2.22	
L	C7/T1	4.76	0.98	0.004**	113.00	3.33	0.533	8.03	1.38	0.054	25.00	6.19	0.141
D		6.81	2.13		112.00	4.36		9.41	2.15		29.10	7.95	

*Also shown are t-test p-values comparing CD and NCD dogs at each cervical vertebral location*.

P–values α <0.05 were considered statistically significant and are flagged with a*. Although the analysis is exploratory, multiple comparisons are considered using a Bonferroni adjusted α <0.0083 with a flag of **.

### Sagittal Plane Measurements

#### Height Ratio

Throughout the cervical spine the height of the dorsal paraspinal musculature increased toward caudal in both breeds. Dachshunds had statistically significant higher mean height ratios than Labrador Retriever at C3/4, C4/5, C7/T1 (*p* = 0.034^*^, *p* = 0.004^**^, *p* = 0.004^**^). Similarly, but not statistically significant, trends were found at C5/C6 and C6/C7 (Figure 4).

### Angle Measurements

There was a statistically significant difference in intervertebral disk angulation to vertebral body length axis at C2/C3 (*p* < 0.001^**^) and C4/C5 (*p* = 0.003^**^) with Labrador Retrievers having the larger angles (Figure 5).

### Transverse Plane Measurements

#### Area Ratio

There were statistically significant differences in dorsal-to-ventral mean musculature area ratios between both breeds, with Dachshunds having the higher ratios, at the level of C3/C4 (*p* < 0.001^**^). At C7/T1 statistical significance was approached (*p* = 0.054) (Figure 6).

#### Mean Ratio of Moments

Both breeds showed an increase in mean ratio of moments from cranial to caudal. There were statistically significant differences in mean ratio of moments between the two groups. Cranially, Labrador Retrievers showed a higher mean ratio of moments compared to Dachshunds, at C2/C3 (*p* = 0.021^*^). In contrast, at C3/C4 there was a statistically significant difference (*p* = 0.001^**^) with Labrador Retrievers having a smaller ratio. Caudally, the ratio of moments increased more severely in Dachshunds, with a higher value at C7/T1, although this did not reach statistical significance (*p* = 0.141) (Figure 7).

## Discussion

In validating our hypothesis we found breed-associated regional differences comparing the dorsal and ventral paraspinal cervical musculature and differences in cervical vertebrae angulation between chondrodystrophic and non-chondrodystrophic dogs.

Previous studies have shown that breed-specific differences in vertebral anatomy can have a significant effect on motion (25, 26). The cervical spine is described as the most flexible region of the canine spine, and within the cervical spine the caudal region, is the one with the greatest range of motion (27). Differences affecting intervertebral disk and endplates morphometry where found within the cervical spine (26). Significant breed-associated differences were also found in canine cervical intervertebral disk-to-vertebral body area and length ratios (28). All these breed specific dissimilarities may play a role in the regional development of cervical spine diseases in predisposed groups of dogs.

To the authors' knowledge, no prior reports have exclusively assessed paraspinal musculature to detect differences as a potential risk factor that might predispose certain breeds to cervical IVDD. Degeneration of the IVD is the fundamental process that lies at the origin of IVDD. Due to these degenerative changes, the NP loses the ability to absorb and maintain water and, thereby, to function as a hydraulic cushion (2, 3, 29–31). The NP is surrounded ventrally, dorsally and laterally by the AF (1, 32, 33). The fibers of the AF provide reinforcement when the healthy IVD is twisted (axial rotation), bent (flexion/extension), and/or compressed (axial compression), with the inner and outer AF mainly resisting compressive and tensile forces, respectively (34–36).

Consequently, more of the compressive forces exerted by the paraspinal muscles, which are normally resisted by the hydrated NP, are taken by AF (37–39). As a result, the AF shows a compensatory increase in functional size (37, 40–42). However, the AF is not built to resist compressive forces, and the increase in functional size consists of a biomechanically inferior matrix (2, 3, 29). As a result, the AF becomes stiffer and weaker leading to structural failure, impeding the AF to resist tensile forces and to contain the NP. Since the dorsal AF is 2 to 3 times thinner than the ventral AF, the dorsal side is usually where the AF shows structural failure and IVD displacement.

The interactions between the musculature, vertebrae and the soft-tissue structures as a motion segment are complex. The paraspinal musculature represents one possible influencing factor for the development of IVDD. The imbalance of dorsal and ventral musculature could lead to an earlier degeneration of the disk. Or, if there is already a degenerated disk (due to other reasons), the imbalance of the paraspinal musculature might also induce variable rates of IVDD at different sites.

The generally higher dorsal-to-ventral ratios in height, area measurements, as well as dorsal-to-ventral moment ratios observed in this study are representative of the stronger developed cervical dorsal paraspinal musculature. However, the statistically significant difference in ratio of moments detected at C2/C3, with Dachshunds having the lower ratio compared to Labrador Retrievers, may play a biomechanical role for development of IVDD at these sites in CD. The smaller ratio is indicative of a relatively stronger ventral paraspinal muscle group especially since the distance between the center of these muscle groups to the center of the disk is smaller than the distance from the center of the dorsal muscle group to this same point. This finding might explain a greater tensile strain on the dorsal aspect of the pathologically less resistant AF at this site that would lead to extrusion in cases of disk degeneration. Contradictorily, this difference could not be identified at C3/C4 even though that site is also frequently affected by IVDD in Dachshunds. Consequently, it is not yet possible to draw a final conclusion, and it is still unclear how adjacent motion segments influence each other and if those transitions could play a role. In addition, the disk angulation to the length axis of the vertebral column observed in the present study may also influence the muscular compressive load bearing applied to the intervertebral disk. Our results showed a steeper conformation (smaller angle, 94°) between C2/C3 in Dachshunds compared to Labrador Retrievers. Steeper intervertebral disk conformation at this location could be an additional influencing biomechanical risk factor for cervical IVDD in CD breeds at C2/C3 because of different load transmission.

In the cervical spine of large breed NCD dogs, the caudal cervical spine, especially C6/C7, is at highest risk for the development of IVD degeneration and displacement (43–45). In the present study, we did not find statistical differences at C6/C7 in vertebrae angulation or in height, area and moment ratios of the paraspinal musculature. However, at C7/T1 the difference in height ratio was statistically significant, which is an uncommon site of intervertebral disk disease in both breeds. It is unclear whether C7/T1 could play a role as an adjacent motion segment and influence the development of IVDD at the site C6/C7. The relevance of this observation is ambiguous. If not clearly associated with cervical muscular anatomy, this high susceptibility has previously been related to the conformation of the facet joints of the caudal cervical spine: due to their shape and conformation, the facet joints of the caudal cervical spine allow considerable more axial rotation and can induce significantly more spinal instability than cranial spinal segments (26, 46). Therefore, the workloads and stresses on the IVDs of the caudal cervical spine may be relatively high, thereby promoting IVD degeneration and displacement at these locations (47).

Several limitations have to be taken in consideration when interpreting the results of the present study. The lack of information about the microstructure of the paraspinal musculature is one major limitation. As the muscle fiber quality is an important factor for the strength of a muscle, this information would have enriched the study. However, histological examination would have required the sampling of muscle tissue, which would have been invasive. Previous reports in human medicine suggest prediction of muscle strength by diffusion tensor imaging (48). Unfortunately, this technique was not accessible for our group during the planning phase of the study. Furthermore, CT-scans were performed on anesthetized dogs, muscle relaxation induced by anesthesia might have influenced the measured values. Since ratios were considered this error might have been minimized. Standardized dorsal recumbency was selected to reduce discrepancies in measurements, even so a minimal percentage of variability is still possible. However, kinematic studies performed on awake dogs would provide more realistic results. Furthermore, due to the fact that the CT-scans were analyzed retrospectively, it was also not possible to create age-matched groups. We therefore presumed that the shape of paraspinal musculature does not change substantially throughout middle age.

The effect of the body condition score on the constitution of paraspinal musculature could not be evaluated even though this could have been beneficial for the study. Body condition scoring was not available in all patients due to the retrospective design of the study and was therefore excluded in the statistical analysis of the data. Further information, which could have been valuable for the study (like head size and neck length), was likewise not available due to the retrospective nature of the study.

The CT-scan was used to perform measurements in the present study, even if an MRI is known to be a more sensitive investigation method when looking at muscles. However, several morphometric studies have outlined the benefits of using CT examinations to analyze anatomical structures of the canine spine (49, 50).

Finally, this study only addressed one possible etiological factor potentially leading to IVDD. Since IVDD has a multifactorial etiology, the collection of a wide range of further information, including other anatomical and morphological risk factors found in previous studies would be necessary. Furthermore, environmental influencing factors, activity level and occupation of the patient (working dog) should be included in the assessment of the real risk of IVDD for a specific patient (51). It would also be interesting to analyse if there are differences within chondrodystrophic breeds that have different vertebrae conformations (e.g. Dachshund vs. French Bulldog).

In conclusion, significant differences have been found in the cervical spine of CD and NCD dogs, regarding paraspinal musculature height and area ratios along with ratio of moments and vertebrae angulation. Those morphometric differences might influence the biomechanics of the intervertebral disk, especially in the upper cervical spine and could therefore play a role in the complex development of IVDD at preferential sites. Further studies are needed to better understand the clinical implications' of differences in paraspinal muscle conformation on intervertebral disk disease.

## Data Availability Statement

The raw data supporting the conclusions of this article will be made available by the authors, without undue reservation.

## Ethics Statement

Ethical review and approval was not required for the animal study because All scans were performed for reasons unrelated to this study. Written informed consent for participation was not obtained from the owners because this was a retrospective study.

## Author Contributions

KH: study design, measurements, generation of the manuscript, and statistical analysis. SK: provision of computed tomographic images and correction of the manuscript. PD and CF: generation of the manuscript. FF: study design and correction of the manuscript. All authors contributed to the article and approved the submitted version.

## Conflict of Interest

The authors declare that the research was conducted in the absence of any commercial or financial relationships that could be construed as a potential conflict of interest.

## References

[1] Hansen HJ. A pathologic-anatomical study on disc degeneration in dog, with special reference to the so-called enchondrosis intervertebralis. Acta Orthop Scand. (1952) 23(Suppl. 11):1–130. 10.3109/ort.1952.23.suppl-11.01 14923291

[2] Ghosh P Taylor TK Braund KG. The variation of the glycosaminoglycans of the canine intervertebral disc with ageing. I. Chondrodystrophoid breed. Gerontology. (1977) 23:87–98. 10.1159/000212177 830252

[3] Ghosh P Taylor TK Braund KG. Variation of the glycosaminoglycans of the intervertebral disc with ageing. II. Non-chondrodystrophoid breed. Gerontology. (1977) 23:99–109. 830253 10.1159/000212178

[4] Harder LK. Diagnostic imaging of changes of the canine intervertebral disc. Tierarztl Prax Ausg K Kleintiere Heimtiere. (2016) 44:359–71. 10.15654/TPK-160468 27658268

[5] Cherrone KL Dewey CW Coates JR Bergman RL. A retrospective comparison of cervical intervertebral disk disease in nonchondrodystrophic large dogs versus small dogs. J Am Anim Hosp Assoc. (2004) 40:316–20. 10.5326/0400316 15238562

[6] Brisson BA. Intervertebral disc disease in dogs. Vet Clin North Am Small Anim Pract. (2010) 40:829–58. 10.1016/j.cvsm.2010.06.001 20732594

[7] Bergknut N Egenvall A Hagman R Gustas P Hazewinkel HAW Meij B. Incidence of intervertebral disk degeneration–related diseases and associated mortality rates in dogs. J Am Vet Med Assoc. (2012) 240:1300–9. 10.2460/javma.240.11.1300 22607596

[8] Bergknut N Smolders LA Grinwis GCM Hagman R Lagerstedt AS Hazewinkel HAW. Intervertebral disc degeneration in the dog. Part 1: anatomy and physiology of the intervertebral disc and characteristics of intervertebral disc degeneration. Vet J. (2013) 195:282–91. 10.1016/j.tvjl.2012.10.024 23177522

[9] Braund KG Taylor TK Ghosh P Sherwood AA. Spinal mobility in the dog. A study in chondrodystrophoid and non-chondrodystrophoid animals. Res Vet Sci. (1977) 22:78–82. 841208

[10] Salomon FV Geyer H Gille U. Anatomie für die Tiermedizin. 2nd ed. Stuttgart: Enke (2008).

[11] Smit TH. The use of a quadruped as an in vivo model for the study of the spine—biomechanical considerations. Eur Spine J. (2002) 11:137–44. 10.1007/s005860100346 11956920 PMC3610505

[12] Fingeroth JM Thomas WB. Advanced in Intervertebral Disc disease in Dogs and Cats. 1st ed. Hoboken, NJ: Wiley Blackwell (2015).

[13] Adams MA Hutton WC. Mechanics of the intervertebral disc. In: Ghosh P, editor. The Biology of the Intervertebral Disc. 1 ed. Boca Raton, FL: CRC Press Inc. (1988) p. 39–733.

[14] White AA III Panjabi MM. Clinical Biomechanics of the Spine. Philadelphia, PA, Toronto, ON: J.B. Lippincott Company (1978).

[15] Putz RL Muller-Gerbl M. The vertebral column-a phylogenetic failure? A theory explaining the function and vulnerability of the human spine. Clin Anat. (1996) 9:205–12. 10.1002/(SICI)1098-2353 8740482

[16] Schilling N Carrier DR. Function of the epaxial muscles in walking, trotting and galloping dogs: implications for the evolution of epaxial muscle function in tetrapods. J Exp Biol. (2010) 213:1490–502. 10.1242/jeb.039487 20400634

[17] Arnold P Forterre F Lang J Fischer MS. Morphological disparity, conservatism, and integration in the canine lower cervical spine: insights into mammalian neck function and regionalization. Mamm Biol. (2015) 81:153–62. 10.1016/j.mambio.2015.09.004

[18] Fischer MS Lilje KE. Dogs in Motion. 2nd ed. Dortmund: VDH Service GmbH (2014).

[19] Marras WS Jorgensen MJ Granata KP Wiand B. Female and male trunk geometry: size and prediction of the spine loading trunk muscles derived from MRI. Clin Biomech (Bristol, Avon). (2001) 16:38–46. 10.1016/s0268-0033(00)00046-2 11114442

[20] Sharir A Milgram J Shahar R. Structural and functional anatomy of the neck musculature of the dog (Canis familiaris). J Anat. (2006) 208:331–51. 10.1111/j.1469-7580.2006.00533.x 16533316 PMC2100244

[21] Budras KD Fricke W Richter R. Atlas der Anatomie des Hundes. 8th ed. Hannover: Schlütersche (2010).

[22] Mihaljević M Kramer M Gomerčić H. CT- und MRT-Atlas. Stuttgart: Parey (2009).

[23] Wisner E Zwingenberger A. Atlas of Small Animal CT and MRI. Hoboken, NJ: Wiley Blackwell (2015).

[24] Alizadeh M Zindl C Allen MJ Knapik GG Fitzpatrick N Marras WS. MRI cross sectional atlas of normal canine cervical musculoskeletal structure. Res Vet Sci. (2016) 109:94–100. 10.1016/j.rvsc.2016.09.009 27892880

[25] Bonelli MA da Costa RC. Clinical and magnetic resonance imaging characterization of cervical spondylomyelopathy in juvenile dogs. J Vet Intern Med. (2019) 33:2160–6. 10.1111/jvim.15602 31469206 PMC6766523

[26] Breit S Kunzel W. Shape and orientation of articular facets of cervical vertebrae C3-C7 in dogs denoting axial rotation ability: an osteological study. Eur J Morphol. (2002) 40:43–51. 10.1076/ejom.40.1.43.13953 12959348

[27] Lang B. Bewegungsmessungen an der Wirbelsäule von Hund und Katze (thesis). Veterinary Medicine, Justus Liebig University, Giessen, Germany (1972).

[28] Düver P Precht C Fosgate G Forterre F Hettlich B. Cervical intervertebral disk to vertebral body ratios of different dog breeds based on sagittal magnetic resonance imaging. Front Vet Sci. (2018) 5:248. 10.3389/fvets.2018.00248 30345279 PMC6182047

[29] Ghosh P Taylor TK Braund KG Larsen LH. The collagenous and non-collagenous protein of the canine intervertebral disc and their variation with age, spinal level and breed. Gerontology. (1976) 22:124–34. 1261806 10.1159/000212129

[30] Gillett NA Gerlach R Cassidy JJ Brown SA. Age-related changes in the beagle spine. Acta Orthop Scand. (1988) 59:503–7. 10.3109/17453678809148772 3188853

[31] Adams MA Roughley PJ. What is intervertebral disc degeneration, and what causes it? Spine (Philadelphia, Pa 1976). (2006) 31:2151–61. 10.1097/01.brs.0000231761.73859.2c 16915105

[32] Cassidy JJ Hiltner A Baer E. Hierarchical structure of the intervertebral disc. Connect Tissue Res. (1989) 23:75–88. 10.3109/03008208909103905 2632144

[33] Samuelson DA. Cartilage and bone. In: Samuelson DA, editor. Textbook of Veterinary Histology. St. Louis, MO: Saunders Elsevier. (2007) p. 100–29.

[34] Hukins DWL. Disc structure and function. In: Ghosh P, editor. The Biology of the Intervertebral Disc. 1st ed. Boca Raton, FL: CRC Press, Inc. (1988) p. 1–38.

[35] Roughley PJ. Biology of intervertebral disc aging and degeneration: involvement of the extracellular matrix. Spine (Phila Pa 1976). (2004) 29:2691–9. 10.1097/01.brs.0000146101.53784.b1 15564918

[36] Setton LA Chen J. Mechanobiology of the intervertebral disc and relevance to disc degeneration. J Bone Joint Surg Am. (2006) 88(Suppl 2):52–7. 10.2106/JBJS.F.00001 16595444

[37] McNally DS Adams MA. Internal intervertebral disc mechanics as revealed by stress profilometry. Spine (Phila Pa 1976). (1992) 17:66–73. 10.1097/00007632-199201000-00011 1536017

[38] McNally DS Shackleford IM Goodship AE Mulholland RC. In vivo stress measurement can predict pain on discography. Spine (Phila Pa 1976). (1996) 21:2580–7. 10.1097/00007632-199611150-00007 8961445

[39] Adams MA McMillan DW Green TP Dolan P. Sustained loading generates stress concentrations in lumbar intervertebral discs. Spine (Phila Pa 1976). (1996) 21:434–8. 10.1097/00007632-199602150-00006 8658246

[40] Adams MA McNally DS Dolan P. 'Stress' distributions inside intervertebral discs. The effects of age and degeneration. J Bone Joint Surg Br. (1996) 78:965–72. 10.1302/0301-620x78b6.1287 8951017

[41] Cole TC Ghosh P Taylor TK. Variations of the proteoglycans of the canine intervertebral disc with ageing. Biochim Biophys Acta. (1986) 880:209–19. 10.1016/0304-4165(86)90082-6 3080032

[42] Johnson JA da Costa RC Allen MJ. Micromorphometry and cellular characteristics of the canine cervical intervertebral discs. J Vet Intern Med. (2010) 24:1343–9. 10.1111/j.1939-1676.2010.0613.x 20946372

[43] Da Costa RC Parent JM Partlow G Dobson H Holmberg DL Lamarre J. Morphologic and morphometric magnetic resonance imaging features of Doberman Pinschers with and without clinical signs of cervical spondylomyelopathy. Am J Vet Res. (2006) 67:1601–12. 10.2460/ajvr.67.9.1601 16948609

[44] Da Costa RC. Cervical spondylomyelopathy (wobbler syndrome) in dogs. Vet Clin North Am Small Anim Pract. (2010) 40:881–913. 10.1016/j.cvsm.2010.06.003 20732597

[45] De Decker S Gielen IM Duchateau L Van Soens I Bavegems V Bosmans T. Low-field magnetic resonance imaging findings of the caudal portion of the cervical region in clinically normal Doberman Pinschers and Foxhounds. Am J Vet Res. (2010) 71:428–34. 10.2460/ajvr.71.4.428 20367050

[46] Johnson JA da Costa RC Allen MJ. Kinematics of the cranial and caudal cervical spine in large breed dogs. In: American College of Veterinary Internal Medicine Forum Proceedings. Lakewood, CO (2010).

[47] Farfan HF Cossette JW Robertson GH Wells RV Kraus H. The effects of torsion on the lumbar intervertebral joints: the role of torsion in the production of disc degeneration. J Bone Joint Surg Am. (1970) 52:468–97. 5425641

[48] Klupp E Cervantes B Schlaeger S Inhuber S Kreuzpointer F Schwirtz A. Paraspinal muscle dti metrics predict muscle strength. J Magn Reson Imaging. (2019) 50:816–23. 10.1002/jmri.26679 30723976 PMC6767405

[49] Jones JC Cartee RE Bartels JE. Computed tomographic anatomy of the canine lumbosacral spine. Vet Radiol Ultrasound. (1995) 36:91–9. 10.1111/j.1740-8261.1995.tb00223.x

[50] Drees R; Dennison SE Keuler NS Schwarz T. Computed tomographic imaging protocol for the canine cervical and lumbar spine. Vet Radiol Ultrasound. (2009) 50:74–9. 10.1111/j.1740-8261.2008.01493.x 19241758

[51] Barandun MA Bult S Demierre S Vidondo B Forterre F. Colder temperatures influence acute onset canine intervertebral disc extrusion. Front Vet Sci. (2020) 7:175. 10.3389/fvets.2020.00175 32318591 PMC7154144

